# Retraction Note: OCT4 as a target of miR-34a stimulates p63 but inhibits p53 to promote human cell transformation

**DOI:** 10.1038/s41419-022-04665-4

**Published:** 2022-03-09

**Authors:** W. L. Ng, G. Chen, M. Wang, H. Wang, M. Story, J. W. Shay, X. Zhang, J. Wang, A. R. M. R. Amin, B. Hu, F. A. Cucinotta, Y. Wang

**Affiliations:** 1 Department of Radiation Oncology, Emory University School of Medicine, Winship Cancer Institute of Emory University, Atlanta, GA USA; 2 Division of Space Life Sciences, Universities Space Research Association, Houston, TX USA; 3 Department of Radiation Oncology, UT Southwestern Medical Center, Dallas, TX USA; 4 Department of Cell Biology, UT Southwestern Medical Center, Dallas, TX USA; 5 Department of Hematology and Oncology, Emory University School of Medicine, Winship Cancer Institute of Emory University, Atlanta, GA USA; 6 Department of Medical Molecular Biology, Beijing Institute of Biotechnology, Beijing, China; 7 Department of Health Physics and Diagnostic Sciences, University of Nevada, Las Vegas, NV USA

Retraction to: *Cell Death Dis* 10.1038/cddis.2013.563 published online 23 January 2014

The Editors-in-Chief have retracted this article because concerns have been raised about the reliability of the presented data. An investigation by the US Office of Research Integrity has concluded that western blot images for total protein expression in human cell lines subject to gene depletion and/or overexpression were produced by reusing immunoblot bands and relabeling them to represent different experiments in seven figure panels in five papers and one grant application. In this article, this affects Fig. 5B, representing p53 and p63 levels in human lung epithelial cells with or without gene depletion.

Authors M. Story and J. W. Shay agree to this retraction. Authors Y. Wang and W. L. Ng do not agree to this retraction. Authors A. R. M. R. Amin and F. A. Cucinotta have not responded to any correspondence from the editor or publisher about this retraction. The Publisher has not been able to obtain a current email address for authors G. Chen, M. Wang, H. Wang, X. Zhang, J. Wang and B. Hu.

